# Supplementary material for: Hot spot prediction in protein-protein interactions by an ensemble system
Source: BMC Syst Biol. 2018 Dec 31;12(Suppl 9):132. doi: 10.1186/s12918-018-0665-8 (PMC6311905; doi:10.1186/s12918-018-0665-8)
Supplement: Supplementary file 2 — All relevant MATLAB code. A simple Matlab implement of our predictor. (ZIP 19,742 kb) [file 12918_2018_665_MOESM2_ESM.zip › Additional file 2/Read ASA/ASA website/dbMPIKT/4V0MB.html]

 NetSurfP - Protein Surface Accessibility and Secondary Structure Predictions

  

|  |  |  |
| --- | --- | --- |
|  |  | NetSurfP - Protein Surface Accessibility and Secondary Structure Predictions  Technical University of Denmark |

  


---

```
# For publication of results, please cite:
# A generic method for assignment of reliability scores applied to solvent accessibility predictions.
# Bent Petersen, Thomas Nordahl Petersen, Pernille Andersen, Morten Nielsen and Claus Lundegaard
# BMC Structural Biology 2009, 9:51 doi:10.1186/1472-6807-9-51
#
# Column 1: Class assignment - B for buried or E for Exposed - Threshold: 25% exposure, but not based on RSA
# Column 2: Amino acid
# Column 3: Sequence name
# Column 4: Amino acid number
# Column 5: Relative Surface Accessibility - RSA
# Column 6: Absolute Surface Accessibility
# Column 7: Z-fit score for RSA prediction
# Column 8: Probability for Alpha-Helix
# Column 9: Probability for Beta-strand
# Column 10: Probability for Coil
E M  4V0MB                  1    0.738 147.694   0.130   0.003   0.003   0.994
E V  4V0MB                  2    0.452  69.411  -0.989   0.019   0.141   0.840
E H  4V0MB                  3    0.471  85.711  -1.007   0.020   0.205   0.775
E E  4V0MB                  4    0.688 120.176  -0.573   0.019   0.141   0.840
E S  4V0MB                  5    0.625  73.262  -1.220   0.018   0.047   0.935
E N  4V0MB                  6    0.478  70.023  -0.095   0.018   0.019   0.964
E P  4V0MB                  7    0.370  52.489  -0.852   0.058   0.017   0.925
E N  4V0MB                  8    0.608  89.084  -1.145   0.058   0.017   0.925
E D  4V0MB                  9    0.446  64.341  -0.991   0.113   0.087   0.800
B Y  4V0MB                 10    0.241  51.587  -0.472   0.118   0.150   0.732
B A  4V0MB                 11    0.289  31.892  -1.427   0.064   0.216   0.721
E A  4V0MB                 12    0.376  41.380  -1.528   0.064   0.216   0.721
E G  4V0MB                 13    0.356  28.017  -1.900   0.056   0.142   0.802
E D  4V0MB                 14    0.495  71.272  -1.048   0.056   0.142   0.802
E A  4V0MB                 15    0.381  41.997  -1.754   0.052   0.084   0.864
E G  4V0MB                 16    0.470  37.013  -2.242   0.052   0.084   0.864
E N  4V0MB                 17    0.490  71.780  -1.031   0.052   0.084   0.864
B A  4V0MB                 18    0.311  34.316  -1.910   0.052   0.084   0.864
E S  4V0MB                 19    0.561  65.808  -1.017   0.056   0.142   0.802
E G  4V0MB                 20    0.334  26.246  -1.980   0.064   0.216   0.721
E R  4V0MB                 21    0.427  97.737  -0.153   0.069   0.386   0.545
B Y  4V0MB                 22    0.261  55.818  -0.414   0.074   0.484   0.442
E T  4V0MB                 23    0.340  47.116  -0.699   0.069   0.386   0.545
E T  4V0MB                 24    0.516  71.555  -0.145   0.066   0.296   0.638
E G  4V0MB                 25    0.344  27.041  -1.895   0.056   0.142   0.802
E S  4V0MB                 26    0.538  63.018  -2.264   0.018   0.088   0.893
E N  4V0MB                 27    0.637  93.198  -1.487   0.018   0.047   0.935
E G  4V0MB                 28    0.431  33.896  -1.817   0.018   0.047   0.935
E I  4V0MB                 29    0.381  70.485  -0.438   0.018   0.047   0.935
B P  4V0MB                 30    0.307  43.535  -0.557   0.018   0.047   0.935
E P  4V0MB                 31    0.348  49.410  -1.663   0.018   0.088   0.893
E M  4V0MB                 32    0.316  63.192  -1.430   0.019   0.141   0.840
B L  4V0MB                 33    0.207  37.938   0.136   0.019   0.141   0.840
E P  4V0MB                 34    0.508  72.142  -0.947   0.052   0.084   0.864
E S  4V0MB                 35    0.500  58.635  -0.561   0.053   0.043   0.903
E V  4V0MB                 36    0.593  91.159  -0.982   0.058   0.017   0.925
E R  4V0MB                 37    0.655 150.064   0.109   0.113   0.043   0.844
E S  4V0MB                 38    0.380  44.513  -0.038   0.118   0.150   0.732
E V  4V0MB                 39    0.335  51.459   0.955   0.216   0.235   0.548
B W  4V0MB                 40    0.087  20.827   0.086   0.231   0.330   0.439
B L  4V0MB                 41    0.088  16.040   0.298   0.352   0.332   0.316
E D  4V0MB                 42    0.294  42.337   0.018   0.321   0.252   0.427
B A  4V0MB                 43    0.092  10.116  -0.866   0.321   0.252   0.427
B F  4V0MB                 44    0.155  31.068  -0.426   0.231   0.330   0.439
E N  4V0MB                 45    0.354  51.782  -0.303   0.216   0.235   0.548
B D  4V0MB                 46    0.177  25.520  -0.602   0.113   0.087   0.800
E P  4V0MB                 47    0.384  54.475  -0.378   0.113   0.043   0.844
B V  4V0MB                 48    0.234  35.997  -1.386   0.184   0.043   0.773
B A  4V0MB                 49    0.149  16.365  -1.024   0.184   0.043   0.773
E G  4V0MB                 50    0.296  23.335  -0.224   0.113   0.043   0.844
B I  4V0MB                 51    0.058  10.711  -0.021   0.118   0.150   0.732
B S  4V0MB                 52    0.287  33.636  -0.374   0.125   0.227   0.648
B A  4V0MB                 53    0.103  11.406  -0.509   0.125   0.227   0.648
B Y  4V0MB                 54    0.138  29.491   0.104   0.118   0.150   0.732
B T  4V0MB                 55    0.244  33.801  -0.959   0.113   0.043   0.844
B P  4V0MB                 56    0.269  38.242  -0.713   0.053   0.043   0.903
B C  4V0MB                 57    0.052   7.343  -0.762   0.052   0.084   0.864
B V  4V0MB                 58    0.084  12.849  -0.410   0.064   0.216   0.721
B H  4V0MB                 59    0.094  17.171  -2.007   0.022   0.552   0.426
B T  4V0MB                 60    0.074  10.250  -0.460   0.023   0.655   0.322
B C  4V0MB                 61    0.031   4.409  -0.580   0.023   0.655   0.322
B N  4V0MB                 62    0.173  25.342  -0.632   0.023   0.655   0.322
B L  4V0MB                 63    0.041   7.470  -0.144   0.022   0.552   0.426
E F  4V0MB                 64    0.290  58.123   0.009   0.021   0.451   0.528
B G  4V0MB                 65    0.257  20.210  -1.230   0.020   0.205   0.775
E D  4V0MB                 66    0.408  58.735  -0.905   0.018   0.047   0.935
E G  4V0MB                 67    0.290  22.807  -1.321   0.018   0.047   0.935
E E  4V0MB                 68    0.404  70.631   0.136   0.020   0.205   0.775
B N  4V0MB                 69    0.068   9.911   0.347   0.021   0.451   0.528
B R  4V0MB                 70    0.169  38.632   1.181   0.023   0.655   0.322
B L  4V0MB                 71    0.028   5.182   1.008   0.011   0.918   0.071
B V  4V0MB                 72    0.034   5.272   0.631   0.006   0.962   0.032
B I  4V0MB                 73    0.030   5.569   0.271   0.011   0.918   0.071
B A  4V0MB                 74    0.050   5.521  -0.093   0.018   0.846   0.136
B D  4V0MB                 75    0.208  29.958   0.090   0.021   0.451   0.528
E E  4V0MB                 76    0.358  62.508  -0.364   0.018   0.088   0.893
E D  4V0MB                 77    0.598  86.100   0.496   0.018   0.047   0.935
E R  4V0MB                 78    0.354  81.135   0.355   0.018   0.088   0.893
E K  4V0MB                 79    0.385  79.215   1.182   0.022   0.359   0.619
B L  4V0MB                 80    0.044   8.093   0.559   0.021   0.756   0.223
E K  4V0MB                 81    0.300  61.648   1.381   0.018   0.846   0.136
B I  4V0MB                 82    0.067  12.339  -0.606   0.018   0.846   0.136
B W  4V0MB                 83    0.139  33.333   0.304   0.018   0.846   0.136
E K  4V0MB                 84    0.489 100.649   0.436   0.021   0.451   0.528
E G  4V0MB                 85    0.426  33.566  -1.436   0.019   0.141   0.840
B T  4V0MB                 86    0.282  39.127  -0.743   0.053   0.043   0.903
E Q  4V0MB                 87    0.535  95.551  -0.072   0.118   0.150   0.732
B K  4V0MB                 88    0.220  45.316   0.719   0.216   0.235   0.548
E A  4V0MB                 89    0.355  39.099  -0.666   0.231   0.330   0.439
E S  4V0MB                 90    0.402  47.079  -0.450   0.321   0.252   0.427
E E  4V0MB                 91    0.407  71.190  -1.030   0.307   0.165   0.527
B H  4V0MB                 92    0.265  48.149   0.134   0.199   0.152   0.649
E P  4V0MB                 93    0.507  71.929  -0.404   0.191   0.086   0.723
B L  4V0MB                 94    0.103  18.786  -0.325   0.113   0.043   0.844
E L  4V0MB                 95    0.335  61.320  -0.747   0.115   0.016   0.868
E D  4V0MB                 96    0.577  83.218  -1.922   0.058   0.017   0.925
B T  4V0MB                 97    0.205  28.489   0.649   0.115   0.016   0.868
B P  4V0MB                 98    0.109  15.510  -0.689   0.052   0.084   0.864
B V  4V0MB                 99    0.069  10.605  -0.436   0.074   0.484   0.442
B A  4V0MB                100    0.038   4.199  -0.880   0.021   0.756   0.223
B I  4V0MB                101    0.032   5.902   0.088   0.018   0.846   0.136
B C  4V0MB                102    0.032   4.479   0.239   0.011   0.918   0.071
B S  4V0MB                103    0.042   4.911  -0.137   0.011   0.918   0.071
B Y  4V0MB                104    0.045   9.681   0.697   0.011   0.918   0.071
B I  4V0MB                105    0.116  21.515   0.391   0.018   0.846   0.136
B S  4V0MB                106    0.140  16.467  -0.501   0.022   0.552   0.426
E E  4V0MB                107    0.308  53.790  -0.140   0.019   0.141   0.840
E N  4V0MB                108    0.567  82.980  -0.853   0.018   0.047   0.935
E T  4V0MB                109    0.563  78.088  -2.171   0.018   0.019   0.964
E A  4V0MB                110    0.445  49.006  -1.161   0.018   0.019   0.964
E P  4V0MB                111    0.434  61.627  -1.014   0.018   0.047   0.935
E R  4V0MB                112    0.408  93.501  -0.171   0.018   0.088   0.893
B L  4V0MB                113    0.182  33.343   0.507   0.019   0.141   0.840
B P  4V0MB                114    0.057   8.074   0.084   0.021   0.279   0.699
B A  4V0MB                115    0.027   2.931   0.126   0.023   0.655   0.322
B L  4V0MB                116    0.029   5.255   0.451   0.018   0.846   0.136
B A  4V0MB                117    0.018   1.962   0.785   0.011   0.918   0.071
B V  4V0MB                118    0.022   3.412   0.880   0.011   0.918   0.071
B A  4V0MB                119    0.022   2.446   0.200   0.018   0.846   0.136
B A  4V0MB                120    0.037   4.121  -0.601   0.022   0.552   0.426
B G  4V0MB                121    0.061   4.840  -0.696   0.018   0.088   0.893
B S  4V0MB                122    0.168  19.678  -1.177   0.058   0.017   0.925
B H  4V0MB                123    0.099  18.026  -0.330   0.125   0.227   0.648
B I  4V0MB                124    0.029   5.346   0.342   0.169   0.612   0.219
B Y  4V0MB                125    0.080  17.032   0.284   0.172   0.690   0.138
B I  4V0MB                126    0.057  10.527   0.365   0.172   0.690   0.138
B Y  4V0MB                127    0.079  16.946   0.325   0.159   0.516   0.325
E R  4V0MB                128    0.335  76.761   0.482   0.135   0.317   0.548
E N  4V0MB                129    0.425  62.220  -0.918   0.052   0.084   0.864
B L  4V0MB                130    0.177  32.445   0.007   0.018   0.047   0.935
E R  4V0MB                131    0.342  78.272   0.459   0.019   0.141   0.840
B P  4V0MB                132    0.240  33.999  -0.767   0.020   0.205   0.775
B Y  4V0MB                133    0.221  47.270   0.041   0.021   0.279   0.699
B Y  4V0MB                134    0.104  22.139  -0.299   0.021   0.451   0.528
E K  4V0MB                135    0.345  70.987  -0.086   0.021   0.451   0.528
B F  4V0MB                136    0.119  23.863   0.090   0.021   0.451   0.528
B V  4V0MB                137    0.239  36.719  -0.245   0.021   0.279   0.699
B L  4V0MB                138    0.123  22.521   0.194   0.004   0.085   0.910
B P  4V0MB                139    0.193  27.330  -0.512   0.005   0.015   0.979
E P  4V0MB                140    0.498  70.638  -1.175   0.005   0.015   0.979
E E  4V0MB                141    0.359  62.630  -0.720   0.005   0.015   0.979
E N  4V0MB                142    0.680  99.567  -0.688   0.005   0.045   0.951
B V  4V0MB                143    0.257  39.562  -0.266   0.018   0.019   0.964
E N  4V0MB                144    0.567  82.965   0.534   0.005   0.015   0.979
E T  4V0MB                145    0.664  92.083   0.434   0.600   0.003   0.397
E E  4V0MB                146    0.495  86.424   0.788   0.782   0.003   0.216
B E  4V0MB                147    0.125  21.750   0.089   0.923   0.002   0.076
E Q  4V0MB                148    0.365  65.225   1.351   0.970   0.001   0.030
E D  4V0MB                149    0.532  76.690   0.999   0.970   0.001   0.030
B I  4V0MB                150    0.082  15.207   0.872   0.970   0.001   0.030
B W  4V0MB                151    0.067  16.017   0.504   0.970   0.001   0.030
E Q  4V0MB                152    0.489  87.371   1.799   0.970   0.001   0.030
E K  4V0MB                153    0.452  92.915   1.493   0.923   0.002   0.076
B V  4V0MB                154    0.087  13.449   0.625   0.858   0.002   0.139
E M  4V0MB                155    0.420  84.102   0.315   0.600   0.003   0.397
E E  4V0MB                156    0.571  99.719  -0.327   0.246   0.004   0.750
E G  4V0MB                157    0.746  58.710  -0.186   0.016   0.005   0.979
E E  4V0MB                158    0.697 121.696   0.086   0.003   0.003   0.994
B I  4V0MB                159    0.115  21.238   0.586   0.018   0.019   0.964
E V  4V0MB                160    0.473  72.746   1.016   0.018   0.047   0.935
B I  4V0MB                161    0.124  22.921  -0.629   0.858   0.002   0.139
E G  4V0MB                162    0.419  32.975  -0.203   0.923   0.002   0.076
E E  4V0MB                163    0.433  75.558   1.506   0.923   0.002   0.076
B A  4V0MB                164    0.041   4.573  -0.406   0.970   0.001   0.030
B V  4V0MB                165    0.171  26.313   0.586   0.970   0.001   0.030
E A  4V0MB                166    0.533  58.704   0.919   0.923   0.002   0.076
B Q  4V0MB                167    0.225  40.185   0.671   0.923   0.002   0.076
B L  4V0MB                168    0.048   8.862   0.126   0.970   0.001   0.030
E T  4V0MB                169    0.467  64.704   1.289   0.923   0.002   0.076
E R  4V0MB                170    0.423  96.775   1.163   0.923   0.002   0.076
B L  4V0MB                171    0.043   7.855   0.561   0.858   0.002   0.139
E Q  4V0MB                172    0.337  60.242   1.171   0.879   0.010   0.111
E V  4V0MB                173    0.530  81.507   0.247   0.879   0.010   0.111
E R  4V0MB                174    0.399  91.417   0.476   0.802   0.014   0.185
E A  4V0MB                175    0.373  41.105  -0.312   0.622   0.015   0.363
E G  4V0MB                176    0.569  44.757  -1.439   0.430   0.016   0.555
E D  4V0MB                177    0.595  85.812  -1.913   0.181   0.016   0.803
E A  4V0MB                178    0.556  61.293  -1.225   0.053   0.043   0.903
E G  4V0MB                179    0.567  44.591  -1.049   0.056   0.142   0.802
B V  4V0MB                180    0.238  36.550   0.161   0.069   0.386   0.545
E V  4V0MB                181    0.472  72.531   0.360   0.074   0.484   0.442
B L  4V0MB                182    0.112  20.452   0.034   0.135   0.317   0.548
B Q  4V0MB                183    0.206  36.720  -0.208   0.199   0.152   0.649
E T  4V0MB                184    0.380  52.734  -0.381   0.717   0.014   0.269
E R  4V0MB                185    0.357  81.753   0.360   0.802   0.014   0.185
B S  4V0MB                186    0.058   6.844  -1.009   0.879   0.010   0.111
B L  4V0MB                187    0.154  28.179  -0.066   0.938   0.007   0.055
E Q  4V0MB                188    0.413  73.815   0.742   0.938   0.007   0.055
B L  4V0MB                189    0.107  19.518   0.076   0.879   0.010   0.111
B M  4V0MB                190    0.120  24.012   0.411   0.802   0.014   0.185
B N  4V0MB                191    0.304  44.476  -0.315   0.430   0.016   0.555
B I  4V0MB                192    0.221  40.885   0.427   0.181   0.016   0.803
E G  4V0MB                193    0.614  48.330  -0.397   0.018   0.019   0.964
E D  4V0MB                194    0.510  73.477   0.458   0.018   0.019   0.964
E P  4V0MB                195    0.485  68.779   0.005   0.694   0.003   0.303
E D  4V0MB                196    0.764 110.092   0.570   0.782   0.003   0.216
E A  4V0MB                197    0.468  51.574   1.179   0.923   0.002   0.076
B K  4V0MB                198    0.133  27.420   0.621   0.923   0.002   0.076
E M  4V0MB                199    0.426  85.343   0.972   0.970   0.001   0.030
E A  4V0MB                200    0.524  57.734   1.173   0.970   0.001   0.030
B F  4V0MB                201    0.106  21.254   1.062   0.988   0.000   0.012
B V  4V0MB                202    0.052   8.069   0.524   0.988   0.000   0.012
E E  4V0MB                203    0.520  90.879   0.931   0.970   0.001   0.030
E H  4V0MB                204    0.420  76.362   1.471   0.970   0.001   0.030
B W  4V0MB                205    0.123  29.654   0.521   0.858   0.002   0.139
E Q  4V0MB                206    0.539  96.194   0.280   0.600   0.003   0.397
E G  4V0MB                207    0.878  69.091   0.075   0.176   0.004   0.820
E Q  4V0MB                208    0.347  61.956   0.241   0.058   0.017   0.925
E P  4V0MB                209    0.441  62.550  -0.059   0.018   0.019   0.964
B L  4V0MB                210    0.095  17.449  -0.625   0.018   0.019   0.964
E V  4V0MB                211    0.413  63.478  -0.154   0.053   0.043   0.903
E A  4V0MB                212    0.578  63.729  -0.881   0.053   0.043   0.903
E T  4V0MB                213    0.298  41.374  -0.239   0.053   0.043   0.903
B T  4V0MB                214    0.239  33.108  -0.950   0.052   0.084   0.864
B V  4V0MB                215    0.123  18.905   0.028   0.135   0.317   0.548
B I  4V0MB                216    0.048   8.972   0.535   0.087   0.683   0.230
B T  4V0MB                217    0.060   8.322   0.548   0.085   0.775   0.140
B C  4V0MB                218    0.019   2.625   0.782   0.072   0.855   0.073
B M  4V0MB                219    0.040   8.064  -0.015   0.085   0.775   0.140
B D  4V0MB                220    0.035   5.044  -1.033   0.085   0.775   0.140
B V  4V0MB                221    0.088  13.541   0.083   0.085   0.775   0.140
B V  4V0MB                222    0.035   5.410   0.144   0.085   0.775   0.140
E K  4V0MB                223    0.273  56.238   0.665   0.169   0.612   0.219
E Q  4V0MB                224    0.273  48.669   0.571   0.216   0.235   0.548
B A  4V0MB                225    0.263  29.016  -0.676   0.199   0.152   0.649
E I  4V0MB                226    0.342  63.196  -0.520   0.191   0.086   0.723
E D  4V0MB                227    0.452  65.133  -1.198   0.113   0.087   0.800
E E  4V0MB                228    0.525  91.735  -0.204   0.058   0.017   0.925
E P  4V0MB                229    0.769 109.093  -1.864   0.018   0.019   0.964
E D  4V0MB                230    0.638  91.878  -0.634   0.016   0.005   0.979
B A  4V0MB                231    0.202  22.304  -1.064   0.018   0.019   0.964
B V  4V0MB                232    0.208  32.000   0.178   0.018   0.047   0.935
B S  4V0MB                233    0.168  19.643  -0.366   0.004   0.138   0.858
B C  4V0MB                234    0.051   7.132   0.927   0.004   0.616   0.381
B L  4V0MB                235    0.026   4.816   0.806   0.001   0.900   0.099
B V  4V0MB                236    0.020   3.089   1.027   0.001   0.900   0.099
B V  4V0MB                237    0.024   3.658   0.961   0.001   0.959   0.040
B G  4V0MB                238    0.022   1.700   0.374   0.018   0.846   0.136
B T  4V0MB                239    0.050   6.866  -0.075   0.022   0.552   0.426
B E  4V0MB                240    0.178  31.079  -0.312   0.021   0.279   0.699
B S  4V0MB                241    0.224  26.311  -0.146   0.018   0.088   0.893
B G  4V0MB                242    0.133  10.436   0.140   0.004   0.138   0.858
B R  4V0MB                243    0.155  35.564   0.556   0.003   0.718   0.279
B I  4V0MB                244    0.021   3.940   1.102   0.001   0.900   0.099
B L  4V0MB                245    0.050   9.100   1.154   0.001   0.959   0.040
B I  4V0MB                246    0.030   5.624   0.323   0.001   0.959   0.040
B L  4V0MB                247    0.045   8.313   0.850   0.002   0.816   0.182
B N  4V0MB                248    0.128  18.681   1.036   0.005   0.336   0.660
E P  4V0MB                249    0.415  58.818   0.100   0.058   0.017   0.925
E A  4V0MB                250    0.568  62.583   0.116   0.181   0.016   0.803
B G  4V0MB                251    0.179  14.048  -0.745   0.268   0.043   0.689
B T  4V0MB                252    0.099  13.704   0.125   0.216   0.235   0.548
E A  4V0MB                253    0.311  34.283   0.332   0.169   0.612   0.219
B I  4V0MB                254    0.097  17.908   0.691   0.172   0.690   0.138
B V  4V0MB                255    0.135  20.780  -0.106   0.085   0.775   0.140
E K  4V0MB                256    0.314  64.508   0.762   0.085   0.775   0.140
E N  4V0MB                257    0.459  67.271   1.361   0.085   0.775   0.140
B I  4V0MB                258    0.121  22.366   0.969   0.018   0.846   0.136
E W  4V0MB                259    0.543 130.688   0.377   0.021   0.756   0.223
B V  4V0MB                260    0.086  13.187   0.089   0.021   0.451   0.528
E G  4V0MB                261    0.485  38.162  -0.253   0.018   0.088   0.893
E I  4V0MB                262    0.377  69.726  -1.266   0.018   0.088   0.893
B T  4V0MB                263    0.194  26.852   0.588   0.056   0.142   0.802
B P  4V0MB                264    0.095  13.452   0.289   0.066   0.296   0.638
B A  4V0MB                265    0.107  11.769  -0.643   0.085   0.775   0.140
B M  4V0MB                266    0.052  10.505   0.405   0.011   0.918   0.071
B I  4V0MB                267    0.028   5.161   0.715   0.011   0.918   0.071
B A  4V0MB                268    0.090   9.962   0.448   0.011   0.918   0.071
B V  4V0MB                269    0.030   4.534  -0.598   0.011   0.918   0.071
B Q  4V0MB                270    0.099  17.699   0.310   0.002   0.816   0.182
B G  4V0MB                271    0.059   4.643  -1.960   0.004   0.616   0.381
B E  4V0MB                272    0.167  29.175  -0.387   0.003   0.718   0.279
B L  4V0MB                273    0.141  25.835  -0.526   0.003   0.718   0.279
E D  4V0MB                274    0.298  42.956   0.346   0.004   0.420   0.576
B V  4V0MB                275    0.267  41.069  -0.869   0.005   0.262   0.733
E G  4V0MB                276    0.459  36.123  -0.526   0.004   0.138   0.858
B Y  4V0MB                277    0.096  20.537  -0.060   0.004   0.616   0.381
B R  4V0MB                278    0.102  23.381   1.024   0.001   0.900   0.099
B I  4V0MB                279    0.022   4.144   1.159   0.001   0.959   0.040
B T  4V0MB                280    0.031   4.369   0.752   0.001   0.959   0.040
B V  4V0MB                281    0.020   3.074   1.228   0.000   0.983   0.017
B A  4V0MB                282    0.035   3.857  -0.064   0.001   0.959   0.040
B G  4V0MB                283    0.026   2.038   0.369   0.001   0.900   0.099
B R  4V0MB                284    0.226  51.754   0.220   0.005   0.336   0.660
E D  4V0MB                285    0.490  70.667  -0.825   0.005   0.015   0.979
E G  4V0MB                286    0.315  24.775  -1.162   0.005   0.015   0.979
B K  4V0MB                287    0.160  32.871   0.137   0.003   0.718   0.279
B L  4V0MB                288    0.039   7.123  -0.081   0.001   0.900   0.099
B Y  4V0MB                289    0.075  16.006   0.796   0.001   0.959   0.040
B H  4V0MB                290    0.074  13.479   0.057   0.001   0.959   0.040
B I  4V0MB                291    0.039   7.252   0.091   0.001   0.900   0.099
E R  4V0MB                292    0.317  72.479   0.322   0.004   0.616   0.381
E N  4V0MB                293    0.465  68.017  -0.323   0.004   0.138   0.858
E G  4V0MB                294    0.472  37.115  -1.277   0.018   0.019   0.964
E E  4V0MB                295    0.657 114.795  -0.627   0.018   0.047   0.935
E L  4V0MB                296    0.380  69.596  -0.780   0.018   0.088   0.893
E S  4V0MB                297    0.464  54.369  -0.560   0.018   0.088   0.893
E Q  4V0MB                298    0.554  99.016   0.694   0.019   0.141   0.840
B T  4V0MB                299    0.231  31.984  -0.117   0.022   0.552   0.426
B I  4V0MB                300    0.227  42.032   0.245   0.021   0.756   0.223
B I  4V0MB                301    0.120  22.274   0.005   0.018   0.846   0.136
E Q  4V0MB                302    0.456  81.442   0.524   0.023   0.655   0.322
B L  4V0MB                303    0.074  13.568   0.313   0.020   0.205   0.775
E E  4V0MB                304    0.586 102.392  -0.964   0.018   0.019   0.964
B A  4V0MB                305    0.261  28.773  -1.389   0.058   0.017   0.925
E Q  4V0MB                306    0.357  63.796   0.186   0.191   0.086   0.723
B P  4V0MB                307    0.093  13.126  -0.256   0.453   0.248   0.299
B V  4V0MB                308    0.126  19.305  -0.522   0.453   0.248   0.299
B G  4V0MB                309    0.152  11.986  -0.957   0.578   0.229   0.194
B L  4V0MB                310    0.070  12.872  -0.198   0.594   0.285   0.121
B V  4V0MB                311    0.094  14.463   0.069   0.504   0.430   0.065
B R  4V0MB                312    0.234  53.678   0.602   0.391   0.466   0.143
B L  4V0MB                313    0.073  13.293   0.417   0.216   0.235   0.548
E A  4V0MB                314    0.448  49.381  -0.244   0.058   0.017   0.925
E K  4V0MB                315    0.352  72.365   0.147   0.018   0.047   0.935
B H  4V0MB                316    0.057  10.314   0.954   0.023   0.655   0.322
B V  4V0MB                317    0.027   4.119   1.064   0.006   0.962   0.032
B A  4V0MB                318    0.031   3.405   0.412   0.006   0.962   0.032
B V  4V0MB                319    0.026   4.073   0.837   0.006   0.962   0.032
B G  4V0MB                320    0.022   1.747   0.701   0.001   0.959   0.040
B C  4V0MB                321    0.037   5.223   0.079   0.001   0.900   0.099
B M  4V0MB                322    0.067  13.447   0.061   0.022   0.552   0.426
E N  4V0MB                323    0.328  48.063   0.104   0.113   0.087   0.800
E D  4V0MB                324    0.403  58.144  -1.026   0.184   0.043   0.773
B V  4V0MB                325    0.201  30.878   0.274   0.231   0.330   0.439
B V  4V0MB                326    0.070  10.744   0.279   0.169   0.612   0.219
B H  4V0MB                327    0.193  35.052   0.561   0.172   0.690   0.138
B A  4V0MB                328    0.111  12.243  -0.672   0.172   0.690   0.138
B Y  4V0MB                329    0.125  26.670   0.282   0.087   0.683   0.230
B T  4V0MB                330    0.182  25.299   0.052   0.022   0.359   0.619
E P  4V0MB                331    0.429  60.861   0.146   0.021   0.279   0.699
E T  4V0MB                332    0.548  76.035   0.866   0.019   0.141   0.840
E G  4V0MB                333    0.340  26.782  -0.946   0.004   0.138   0.858
E H  4V0MB                334    0.491  89.295   0.390   0.004   0.514   0.481
E K  4V0MB                335    0.332  68.354   0.090   0.002   0.816   0.182
E S  4V0MB                336    0.330  38.723  -0.558   0.001   0.900   0.099
B W  4V0MB                337    0.165  39.610  -0.597   0.001   0.900   0.099
E S  4V0MB                338    0.389  45.544   0.288   0.001   0.900   0.099
B L  4V0MB                339    0.064  11.737   0.739   0.001   0.900   0.099
E Y  4V0MB                340    0.450  96.058   0.322   0.003   0.718   0.279
B L  4V0MB                341    0.087  16.021   0.377   0.005   0.336   0.660
E P  4V0MB                342    0.479  68.027  -0.793   0.018   0.088   0.893
B C  4V0MB                343    0.293  41.165  -1.071   0.018   0.047   0.935
E H  4V0MB                344    0.368  66.848   0.063   0.056   0.142   0.802
B I  4V0MB                345    0.067  12.451   0.035   0.022   0.359   0.619
B L  4V0MB                346    0.190  34.844   0.235   0.023   0.655   0.322
B A  4V0MB                347    0.131  14.403  -0.857   0.023   0.655   0.322
B M  4V0MB                348    0.083  16.568  -0.495   0.022   0.552   0.426
B Q  4V0MB                349    0.182  32.523  -0.958   0.022   0.552   0.426
B R  4V0MB                350    0.249  57.113  -0.644   0.022   0.552   0.426
B M  4V0MB                351    0.120  23.952   0.702   0.022   0.552   0.426
E E  4V0MB                352    0.446  77.864  -0.262   0.004   0.420   0.576
B V  4V0MB                353    0.142  21.856  -0.093   0.020   0.205   0.775
E T  4V0MB                354    0.550  76.216  -1.154   0.053   0.043   0.903
E G  4V0MB                355    0.492  38.689  -0.993   0.113   0.043   0.844
E Q  4V0MB                356    0.554  98.998  -1.279   0.184   0.043   0.773
E R  4V0MB                357    0.379  86.699  -0.243   0.113   0.043   0.844
E N  4V0MB                358    0.430  62.937   0.323   0.118   0.150   0.732
B T  4V0MB                359    0.047   6.561   0.484   0.074   0.484   0.442
B K  4V0MB                360    0.183  37.746   1.116   0.085   0.775   0.140
B A  4V0MB                361    0.020   2.248   0.910   0.011   0.918   0.071
B L  4V0MB                362    0.022   4.047   1.291   0.011   0.918   0.071
B I  4V0MB                363    0.015   2.868   1.526   0.006   0.962   0.032
B V  4V0MB                364    0.024   3.735   1.137   0.006   0.962   0.032
B A  4V0MB                365    0.023   2.491   0.201   0.011   0.918   0.071
B L  4V0MB                366    0.059  10.876   0.211   0.085   0.775   0.140
E S  4V0MB                367    0.354  41.524  -0.238   0.020   0.205   0.775
E N  4V0MB                368    0.617  90.285   0.160   0.005   0.045   0.951
E G  4V0MB                369    0.287  22.555  -0.194   0.005   0.045   0.951
E E  4V0MB                370    0.352  61.582   0.577   0.004   0.514   0.481
B V  4V0MB                371    0.054   8.269   0.776   0.001   0.900   0.099
B R  4V0MB                372    0.235  53.838   1.575   0.001   0.959   0.040
B V  4V0MB                373    0.076  11.650  -0.006   0.001   0.959   0.040
B Y  4V0MB                374    0.097  20.836   0.984   0.001   0.900   0.099
E N  4V0MB                375    0.329  48.151   0.928   0.004   0.514   0.481
E E  4V0MB                376    0.577 100.872  -0.703   0.004   0.085   0.910
E K  4V0MB                377    0.519 106.820   0.367   0.004   0.085   0.910
E L  4V0MB                378    0.293  53.575   0.448   0.004   0.514   0.481
B L  4V0MB                379    0.127  23.327   0.459   0.004   0.616   0.381
B V  4V0MB                380    0.137  21.072   0.508   0.021   0.756   0.223
B S  4V0MB                381    0.228  26.757  -0.659   0.018   0.846   0.136
B V  4V0MB                382    0.167  25.622   0.267   0.011   0.918   0.071
B H  4V0MB                383    0.108  19.572   0.056   0.011   0.918   0.071
E V  4V0MB                384    0.383  58.821   0.310   0.002   0.816   0.182
B S  4V0MB                385    0.098  11.439   0.164   0.022   0.552   0.426
E P  4V0MB                386    0.410  58.151  -0.249   0.056   0.142   0.802
E N  4V0MB                387    0.548  80.154  -1.264   0.113   0.043   0.844
B P  4V0MB                388    0.279  39.576  -0.632   0.268   0.043   0.689
B V  4V0MB                389    0.101  15.524  -0.757   0.428   0.171   0.402
B T  4V0MB                390    0.203  28.114  -0.125   0.453   0.248   0.299
B A  4V0MB                391    0.076   8.364  -0.620   0.373   0.401   0.226
B L  4V0MB                392    0.055  10.107   0.567   0.391   0.466   0.143
B W  4V0MB                393    0.176  42.256   0.946   0.268   0.505   0.227
B F  4V0MB                394    0.077  15.414  -0.528   0.159   0.516   0.325
B G  4V0MB                395    0.070   5.525  -1.502   0.069   0.386   0.545
B R  4V0MB                396    0.178  40.739  -0.327   0.069   0.386   0.545
B Y  4V0MB                397    0.098  20.878  -0.336   0.069   0.386   0.545
B G  4V0MB                398    0.184  14.512  -0.930   0.064   0.216   0.721
B R  4V0MB                399    0.245  56.151  -0.185   0.056   0.142   0.802
E E  4V0MB                400    0.296  51.694  -0.803   0.056   0.142   0.802
E D  4V0MB                401    0.440  63.375   0.285   0.052   0.084   0.864
B N  4V0MB                402    0.140  20.437  -0.023   0.064   0.216   0.721
B T  4V0MB                403    0.040   5.493  -0.205   0.023   0.655   0.322
B L  4V0MB                404    0.027   4.962   0.759   0.011   0.918   0.071
B L  4V0MB                405    0.024   4.394   0.563   0.011   0.918   0.071
B A  4V0MB                406    0.029   3.185   0.389   0.006   0.962   0.032
B I  4V0MB                407    0.023   4.255   0.729   0.006   0.962   0.032
B T  4V0MB                408    0.052   7.157   0.105   0.011   0.918   0.071
E K  4V0MB                409    0.259  53.338   0.233   0.005   0.336   0.660
E S  4V0MB                410    0.457  53.525  -1.262   0.005   0.045   0.951
B G  4V0MB                411    0.162  12.773  -1.707   0.018   0.047   0.935
B A  4V0MB                412    0.107  11.758  -1.546   0.022   0.552   0.426
B L  4V0MB                413    0.045   8.203   0.480   0.011   0.918   0.071
B D  4V0MB                414    0.081  11.629  -0.069   0.006   0.962   0.032
B I  4V0MB                415    0.041   7.640   0.370   0.006   0.962   0.032
B K  4V0MB                416    0.157  32.336   0.490   0.001   0.959   0.040
B M  4V0MB                417    0.119  23.872   0.715   0.001   0.959   0.040
B L  4V0MB                418    0.084  15.362   0.316   0.001   0.900   0.099
E P  4V0MB                419    0.415  58.846   0.776   0.021   0.756   0.223
B R  4V0MB                420    0.268  61.280   0.101   0.021   0.451   0.528
E T  4V0MB                421    0.482  66.812  -0.600   0.022   0.359   0.619
B A  4V0MB                422    0.225  24.817  -1.402   0.021   0.279   0.699
E N  4V0MB                423    0.567  83.067   0.683   0.021   0.279   0.699
B L  4V0MB                424    0.225  41.179  -0.451   0.019   0.141   0.840
E E  4V0MB                425    0.888 155.169   0.881   0.003   0.003   0.994

Explain the output.  Go back.
```

---
